# The role of IL-6 in the physiologic versus hypertensive blood pressure actions of angiotensin II

**DOI:** 10.14814/phy2.12595

**Published:** 2015-10-20

**Authors:** M Marlina Manhiani, Dale M Seth, Amy K L Banes-Berceli, Ryosuke Satou, L Gabriel Navar, Michael W Brands

**Affiliations:** 1 Department of Physiology, Medical College of GeorgiaAugusta, Georgia; 2 Department of Physiology and Hypertension and Renal Center of Excellence, Tulane UniversityNew Orleans, Louisiana; 3 Department of Biological Sciences, Oakland UniversityRochester, Michigan

**Keywords:** Angiotensin II, blood pressure, IL-6, JAK/STAT, low-salt diet

## Abstract

Angiotensin II (AngII) is a critical physiologic regulator of volume homeostasis and mean arterial pressure (MAP), yet it also is known to induce immune mechanisms that contribute to hypertension. This study determined the role of interleukin-6 (IL-6) in the physiologic effect of AngII to maintain normal MAP during low-salt (LS) intake, and whether hypertension induced by plasma AngII concentrations measured during LS diet required IL-6. IL-6 knockout (KO) and wild-type (WT) mice were placed on LS diet for 7 days, and MAP was measured 19 h/day with telemetry. MAP was not affected by LS in either group, averaging 101 ± 4 and 100 ± 4 mmHg in WT and KO mice, respectively, over the last 3 days. Seven days of ACEI decreased MAP ∼25 mmHg in both groups. In other KO and WT mice, AngII was infused at 200 ng/kg per minute to approximate plasma AngII levels during LS. Surgical reduction of kidney mass and high-salt diet were used to amplify the blood pressure effect. The increase in MAP after 7 days was not different, averaging 20 ± 5 and 22 ± 6 mmHg in WT and KO mice, respectively. Janus Kinase 2 (JAK2)/signal transducer of activated transcription (STAT3) phosphorylation were not affected by LS, but were increased by AngII infusion at 200 and 800 ng/kg per minute. These data suggest that physiologic levels of AngII do not activate or require IL-6 to affect blood pressure significantly, whether AngII is maintaining blood pressure on LS diet or causing blood pressure to increase. JAK2/STAT3 activation, however, is tightly associated with AngII hypertension, even when caused by physiologic levels of AngII.

## Introduction

The involvement of immune mechanisms in hypertension is firmly established (Mattson 2014; Singh et al. 2014; McMaster et al. 2015; Zhang and Crowley 2015), and the angiotensin II (AngII) model of hypertension has contributed significantly to our understanding of these mechanisms (Crowley et al. 2006; Guzik et al. 2007). However, often lost behind the spotlight on AngII hypertension is the appreciation that AngII exerts up to ∼20–30 mmHg of influence on blood pressure to keep it from falling during low-salt (LS) intake (Davis et al. 1974; Hall et al. 1980). The magnitude of that effect on blood pressure is in the range of the blood pressure increase reported in many models of AngII hypertension (Hall et al. 1980; Kawada et al. 2002; Gonzalez-Villalobos et al. 2009; Zhao et al. 2009; Brands et al. 2010). Therefore, this raises the question of whether the inflammatory mechanisms that contribute to AngII hypertension also play a role in the powerful effect of AngII to support blood pressure during LS diet.

One of the potential immune mediators in AngII hypertension is interleukin-6 (IL-6) (Lee et al. 2006; Satou et al. 2008, 2009; Brands et al. 2010; Kirabo et al. 2011). Previous studies reported that AngII hypertension was attenuated in IL-6 knockout (KO) mice (Lee et al. 2006; Brands et al. 2010). However, Chamarthi et al. (Chamarthi et al. 2011) reported that human subjects on LS diet did not have elevated plasma IL-6 levels, despite increased plasma renin activity and the effect of acute AngII infusion to increase plasma IL-6. Similarly, intrarenal angiotensinogen, which is stimulated by AngII in an IL-6-dependent manner (Satou et al. 2008, 2009), is not increased on LS diet (Shao et al. 2013). In addition, we have shown that Janus Kinase 2 (JAK2), which is activated in AngII hypertension (Marrero et al. 1998; Brands et al. 2010; Banes-Berceli et al. 2011) except in IL-6 KO mice (Brands et al. 2010), is not required for maintenance of normal blood pressure during LS intake (Banes-Berceli et al. 2011). Those data suggest that maintenance of normal blood pressure on LS diet does not require IL-6, but that has not been tested. Therefore, the first goal of this study was to test the hypothesis that IL-6 is not required to maintain normal blood pressure during LS intake.

If IL-6 is not required for maintenance of normal blood pressure during LS diet, the next question, given the powerful influence of AngII in that process, is why? The comparability of blood pressure power of AngII on LS diet versus that in many models of AngII hypertension noted above suggests that the magnitude of blood pressure influence alone is not the answer. Therefore, whether AngII is exerting its blood pressure influence to maintain normal blood pressure, that is, preventing it from falling, versus raising pressure above normal, that is, hypertension, could be a factor. Another possibility simply could be the plasma levels of AngII. Therefore, we also tested the role of the blood pressure range under which AngII is operating. Specifically, we determined whether the requirement for IL-6 for AngII to induce ∼25 mmHg of hypertension is due to the fact that blood pressure is above normal. We tested this by inducing hypertension with plasma AngII levels that mimicked those caused by the physiologic response to LS diet. We accomplished that by infusing a very low dose of AngII (200 ng/kg per minute) in wild-type (WT) and IL-6 KO mice with reduced kidney mass (RKM) and on 4% high-salt diet. That combination raised blood pressure significantly, and enabled us to test the role of IL-6 under hypertensive conditions when plasma AngII levels were not different from those measured in animals that were eating LS diet.

## Materials and Methods

Procedures involving animals were approved by the Animal Care and Use Committee of Georgia Regents University. The experiments were conducted in 12- to 14-week-old (23–28 g) male mice from Jackson Laboratories (Bar Harbor, ME). The IL-6 KO mice (B6.129S2-Il6^tm1Kopf^/J) are on a C57BL/6J background, and those were the WT control mice we used. In all mice, biotelemetry transmitter devices (Data Sciences International (DSI), St. Paul, MN, PA-C10) were implanted in the left carotid artery under isoflurane anesthesia using aseptic technique. Mice were transferred to a light- and temperature-controlled room in the animal facilities, and were housed individually in standard mouse cages with tap water and rodent chow available ad libitum. They were given 5–7 days to recover from surgery before Control measurements were made.

### Role of IL-6 during LS diet

After Control measurements on standard rodent chow (0.4% sodium), IL-6 KO and WT mice were placed on LS diet (Teklad custom diet, Envigo, Indianapolis, IN, 0.01% sodium) for 7 days. In order to quantify the amount of AngII-dependent blood pressure support on the LS diet in both groups, all mice then were given an ACEI (lisinopril, 100 mg/L in the drinking water) (Gonzalez-Villalobos et al. 2009) for an additional 7 days before being sacrificed. Mean arterial pressure (MAP) was measured in all mice every day, 19 h/day, by telemetry.

### Influence of blood pressure range in determining the role of IL-6

Mice were assigned randomly to five groups: AngII infusion in WT with RKM, AngII infusion in IL-6 KO with RKM, Vehicle infusion in WT with RKM, Vehicle infusion in IL-6 KO with RKM, and AngII infusion in WT mice with normal kidney mass. Kidney mass was reduced surgically in a two-step procedure. In the first step, the poles of the left kidney were removed surgically via a small flank incision under isoflurane anesthesia. A 4-0 silk ligature was tightened around each pole, serving both to excise the pole and provide hemostasis. Two to 3 weeks later, the right kidney was removed via flank incision during the same surgical procedure in which the DSI biotelemetry device was implanted, resulting in ∼70% surgical reduction in kidney mass. Control measurements were made on LS diet (Harlan-Teklad custom diet, 0.01% sodium), and then Alzet 1002 osmotic minipumps were implanted to infuse Vehicle (saline) or AngII for 14 days at 200 ng/kg per minute, s.c. After the first 7 days of AngII infusion on LS diet, all mice were switched to high-salt diet (Harlan-Teklad custom diet, 4% sodium) for 7 days.

### Plasma AngII concentration and kidney JAK2/STAT3

Additional mice were assigned randomly to five groups: Control WT, LS WT, LS KO, AngII 200 in WT, and AngII 800 in WT. The LS mice were given the same LS diet (Teklad custom diet, 0.01% sodium) used in the other experiments, and the other mice were on standard rodent chow (0.4% sodium). Both AngII groups were infused with AngII using Alzet 1002 osmotic minipumps (DURECT Corporation, Cupertino, CA), at either 200 or 800 ng/kg per minute. After 10–14 days under the respective treatment, arterial blood was sampled from the abdominal aorta in isoflurane-anesthetized mice. Plasma samples were frozen and shipped to Dr. Navar’s laboratory for measurement of plasma AngII concentration (Fox et al. 1992; Imig et al. 1999). Kidneys were removed after blood sampling, quick frozen in liquid nitrogen, stored at −80°C, and shipped to Dr. Banes-Berceli’s laboratory for western blot analysis.

### Analytic methods

#### Blood pressure measurement

Mouse cages were placed individually on Data Sciences receivers, and pulsatile arterial pressure was recorded from 1500 to 1000 h (i.e., 19 h) each day. Analog signals from the transmitters were sampled for 5 sec every 2 min at 500 Hz, and the average of those measurements was recorded as the daily MAP for each animal.

#### Plasma AngII and IL-6 concentrations

For AngII, reconstituted extracts were incubated with a rabbit anti-ANGII serum (Peninsula Laboratories, San Carlos, CA) and ^125^I-radiolabeled AngII (Perkin Elmer Life and Analytic Sciences, Waltham, MA) for 48 h at 4°C. Bound and free ANG peptides were separated by dextran-coated charcoal, and the supernatants were counted on a computer-linked gamma-counter for 3 min. The sensitivity of the AngII assay was 0.79 fmol. For the AngII assays, the specific binding was 78.5% and nonspecific binding was 0.5%. Plasma IL-6 concentrations were measured by enzyme immunoassay (R&D Systems, Minneapolis, MN).

#### Kidney JAK2/STAT3

Briefly, supernatant were separated on sodium dodecyl sulfate-polyacrylamide gels (7.5% SDS-PAGE) and transferred to Immobilon-P membrane. Membranes were blocked and probed overnight (4°C) with primary antibodies raised against the phosphorylated forms of the proteins (pJAK2, JAK2, pSTAT3, STAT3, SHP-1 serine591, SHP-1 tyrosine564, SHP-1; Abcam, Cambridge, MA). Blots were washed, and an anti-rabbit horseradish peroxidase-linked secondary antibody was added for 1 h and incubated with the blots at 4°C. Blots were washed and enhanced chemiluminescence (Super Signals Ultra; Pierce-Thermo Fisher Scientific, Waltham, MA) was used to visualize labeled bands. Blots were stripped and reprobed with the antibody raised against the unphosphorylated forms of the proteins. Beta actin (Cell Signaling, Danvers, MA) was used to ensure equal total protein loading between lanes. Band density was quantified using the program NIH Image.

### Statistical analysis

**Figure 1:**
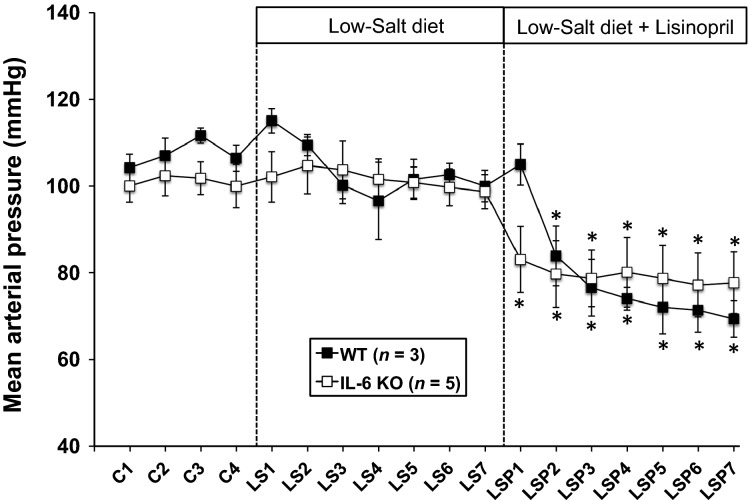
MAP in WT and IL-6 KO mice during a 4-day control period (C) on standard laboratory chow, 7 days on LS diet, and 7 day on LS diet plus the ACEI, LSP, in the drinking water. *P <* *0.05 within-group versus the average MAP over the last 3 days of the LS period. WT, wild-type; KO, IL-6 knockout; LS, low-salt; MAP, mean arterial pressure; LSP, lisinopril.

**Figure 2:**
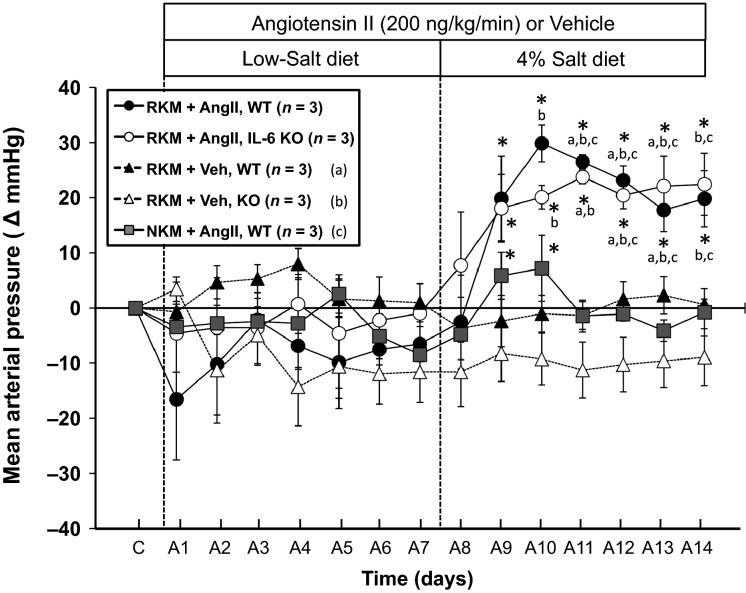
The change in MAP from control (C) MAP in WT and IL-6 KO mice, with RKM or NKM, during 14 days of AngII or Veh infusion – the first 7 days on LS diet and the last 7 days on 4% high-salt diet. *P <* *0.05 within-group versus the average MAP over the last 3 days of the AngII + LS period. (a) *P *<* *0.05 versus RKM + Veh, WT; (b) *P *<* *0.05 versus RKM + Veh, KO; (c) *P *<* *0.05 versus NKM + AngII, WT. MAP, mean arterial pressure; WT, wild-type; KO, IL-6 knockout; RKM, reduced kidney mass; NKM, normal kidney mass; Veh, vehicle; LS, low-salt; AngII, angiotensin II.

Homogeneity of variance was confirmed with *F*-test, and time- and treatment-dependent changes in MAP were analyzed with a two-factor, repeated measures analysis of variance (ANOVA). The control period was averaged to a single Control value, and the last 3 days of the initial 7-day LS period in both MAP experiments (Figs.1, 2) were averaged to single LS values for each group in those respective periods. Significant *F*-values were followed by Dunnett’s test to determine specific within-group differences over time. Significant *F*-values for treatment effect were followed by unpaired *t*-tests (Fig.1) and completely randomized ANOVA plus Scheffe’s test (Fig.2) on each day to determine specific between-group differences on each day. Plasma AngII, and JAK2, STAT3, and SHP-1 data, were analyzed with completely randomized ANOVA. Significance was *P *<* *0.05, and data are expressed as mean ± SEM.

## Results

### Role of IL-6 during LS diet

Figure1 shows that MAP was not different between IL-6 KO and WT mice during Control measurements on standard rodent chow or during 7 days of LS diet. There also was no significant change in blood pressure during that 7-day period. The ACEI, lisinopril, caused a rapid and significant decrease in MAP in both groups that was not different between groups.

### Influence of blood pressure range in determining the role of IL-6

The left side of Figure2 shows that AngII infusion at the very low dose of 200 ng/kg per minute had no significant effect on MAP whether kidney mass was normal or reduced, or whether IL-6 was present or not, when mice were on LS diet. Most mice tended to have a drop in blood pressure on the LS diet following the minipump implantation procedure, but the RKM KO mice with Vehicle (open triangles) remained there even during the second week on 4% salt diet. Baseline MAP in those RKM mice was not different from that in the RKM WT mice with Vehicle (146 ± 2 vs. 144 ± 1 mmHg, respectively), just as there were no differences in baseline MAP in normal kidney mass WT versus IL-6 KO mice as shown in Figure1 and our previous studies (Lee et al. 2006; Brands et al. 2010). However, the RKM WT mice with AngII (black circles) also had a notable drop in MAP after minipump implantation, but this did not affect their ability to respond to 4% salt with a significant increase in MAP. The right side of the figure shows that when mice were switched to 4% high-salt diet, there was only a transient (2 days) rise in MAP in AngII-infused mice with normal kidney mass. Similarly, high-salt diet did not significantly change MAP in vehicle-infused WT or KO mice with RKM. However, high-salt diet increased MAP significantly throughout the 7-day period in AngII-infused RKM mice, and the hypertensive response was not different between WT and IL-6 KO mice.

**Figure 3:**
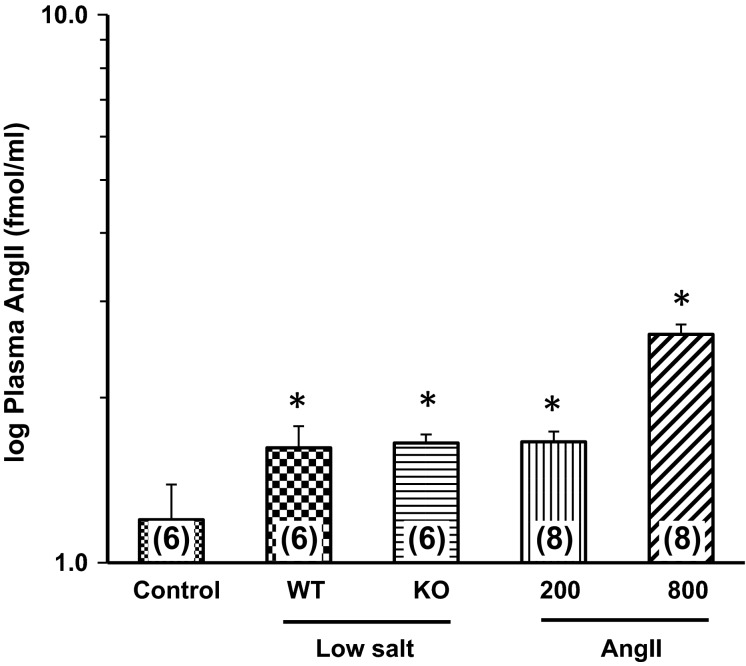
Plasma AngII concentration in WT mice under Control conditions, WT and KO mice on Low-Salt diet, and WT mice infused for 10–14 days with AngII at 200 or 800 ng/kg per minute. Numbers in parentheses = *n*. *P <* *0.05 versus Control. AngII, angiotensin II; WT, wild-type; KO, IL-6 knockout; AngII, Angiotensin II.

Plasma AngII concentration was log-transformed to yield homogeneity of variance, and the data are shown in Figure3. Plasma AngII concentrations in the LS WT, LS KO, and AngII 200 groups averaged 56 ± 18, 47 ± 6, and 51 ± 8 fmol/mL, respectively. These were significantly different than the 23 ± 7 fmol/mL in the Control mice, but not versus each other. A group of mice was infused with AngII at 800 ng/kg per minute to provide a link for the plasma IL-6 response to our previous study of AngII hypertension in mice (Brands et al. 2010), and we also measured plasma AngII concentration in those mice. It increased significantly to 512 ± 130 fmol/mL. That increase was out of proportion to the 200 ng/kg per minute group relative to infusion dose, but we do not have enough data points to accurately analyze the pharmacokinetics. Plasma IL-6 was not detectable in Control (similar to our previous finding) (Brands et al. 2010), LS, or AngII-200 groups, and averaged 22 + 2 pg/mL in mice infused with AngII at 800 ng/kg per minute (similar to the range we reported previously; (Brands et al. 2010).

**Figure 4:**
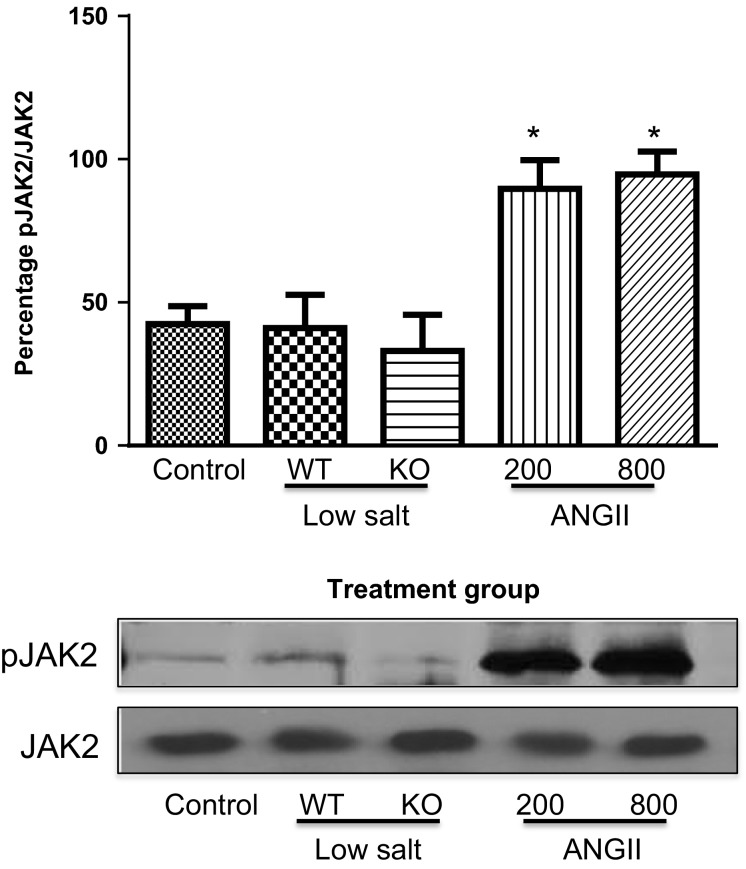
Percentage of pJAK2 to unphosphorylated JAK2 in WT mice under Control conditions, WT and KO mice on Low-Salt diet, and WT mice infused for 10–14 days with AngII at 200 or 800 ng/kg per minute. *P <* *0.05 versus Control. pJAK2, phosphorylated JAK2; WT, wild-type; KO, IL-6 knockout; AngII, angiotensin II.

**Figure 5:**
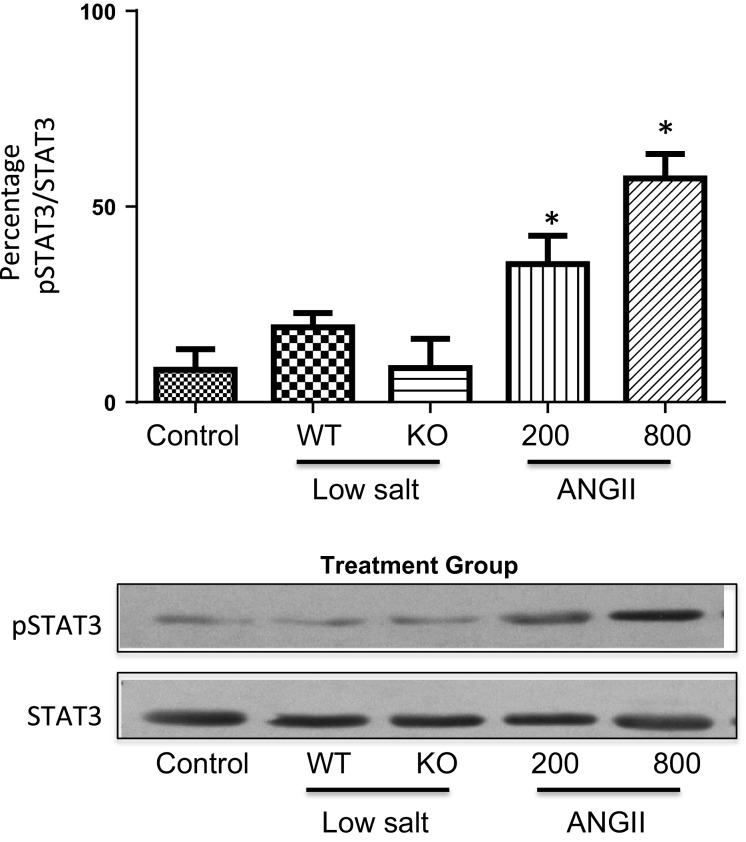
Percentage of pSTAT3 to unphosphorylated STAT3 in WT mice under Control conditions, WT and KO mice on Low-Salt diet, and WT mice infused for 10–14 days with AngII at 200 or 800 ng/kg per minute. Mice in Control and AngII groups were WT mice. *P <* *0.05 versus Control. pSTAT3, phosphorylated STAT3; WT, wild-type; KO, IL-6 knockout; AngII, angiotensin II.

**Figure 6:**
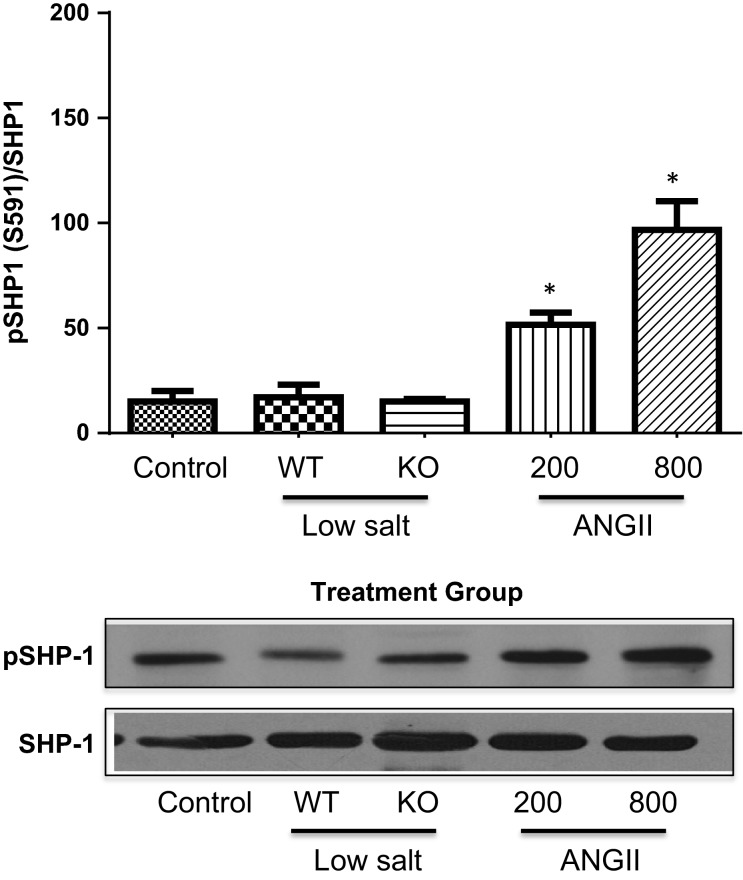
Percentage of pSHP1[S591] to unphosphorylated SHP-1 in WT mice under Control conditions, WT and KO mice on Low-Salt diet, and WT mice infused for 10–14 days with AngII at 200 or 800 ng/kg per minute. *P <* *0.05 versus Control. pSHP1[S591], SHP-1 phosphorylated at serine 591; WT, wild-type; KO, IL-6 knockout; AngII, angiotensin II.

**Figure 7:**
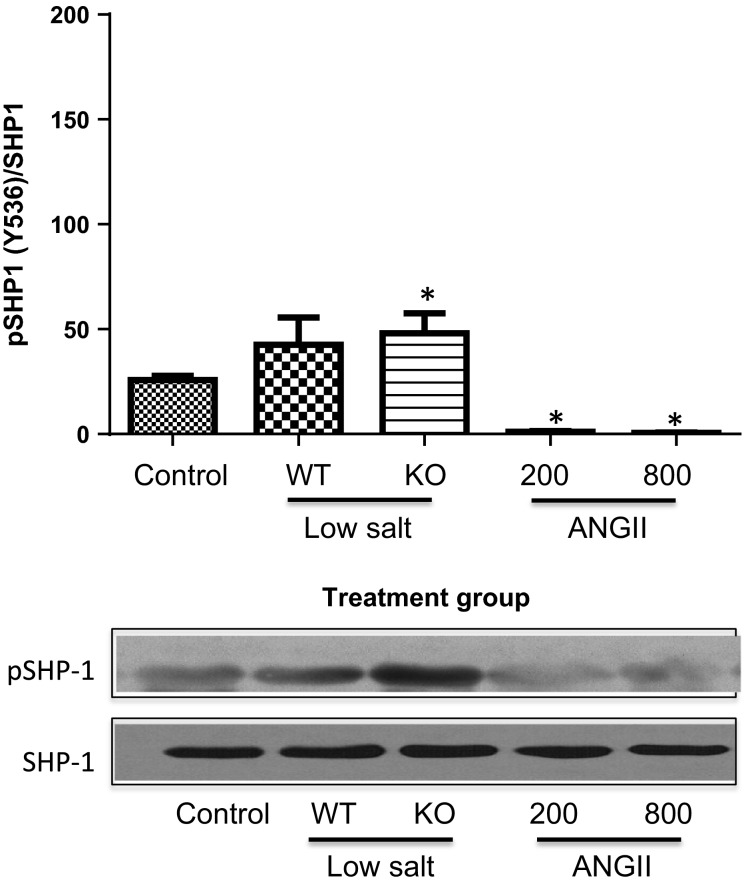
Percentage of pSHP1[Y536] to unphosphorylated SHP-1 in WT mice under Control conditions, WT and KO mice on Low-Salt diet, and WT mice infused for 10–14 days with AngII at 200 or 800 ng/kg per minute. Mice in Control and AngII groups were WT mice. *P <* *0.05 versus Control. pSHP1[Y536], SHP-1 phosphorylated at tyrosine 536; WT, wild-type; KO, IL-6 knockout; AngII, angiotensin II.

Figure4 shows that the phosphorylated tyrosine levels of JAK2 (pJAK2) increased significantly only in the mice infused with AngII, and this was accompanied by tyrosine phosphorylation of the signal transducer of activated transcription (STAT)3 (Fig.5). Tyrosine phosphorylation of these proteins leads to their activation. This is consistent with our previous studies that demonstrated a link between AngII hypertension and activation of the JAK2/STAT3 pathway (Brands et al. 2010; Banes-Berceli et al. 2011). Furthermore, inhibition of JAK2 with AG490 prevented AngII-induced hypertension and decreased the activity of STAT3 (Brands et al. 2010; Banes-Berceli et al. 2011). To test further the role and regulation of the JAK2/STAT3 pathway under these conditions, the activation of SHP-1 was determined. SHP-1 has been shown to dephosphorylate JAK2 and terminate the AngII-induced JAK/STAT cascade (Marrero et al. 1998). Figures6, 7 show increased inhibitory serine (S591) and decreased activating tyrosine (Y564) phosphorylation, respectively, of SHP-1 in both of the AngII-infused groups, suggesting inhibition of SHP-1. This association of inhibitory phosphorylation of SHP-1 and activated JAK2 and STAT3 is consistent with previous reports (Marrero et al. 1998; Brands et al. 2010; Banes-Berceli et al. 2011), and strengthens the association of JAK2/STAT3 activation only in the AngII-infused groups.

## Discussion

The main finding from this study is that the effect of AngII to support blood pressure on LS diet does not require IL-6. Plasma IL-6 was not measurable and JAK2/STAT3 were not activated in mice on LS diet. IL-6 KO mice maintained blood pressure unchanged at control levels for 7 days of sodium-deficient diet. The marked drop in blood pressure induced by subsequent administration of lisinopril showed that AngII indeed was supporting blood pressure significantly during LS diet. Moreover, the responses of the KO mice to LS diet and lisinopril were not different from WT mice, and there was no evidence that plasma AngII concentration was different between WT and KO mice on LS diet. These data provide evidence that the physiologic actions of AngII on chronic blood pressure control do not require IL-6.

The clinical significance of hypertension and the utility of AngII hypertension as an experimental model of hypertension understandably divert routine awareness away from the physiological actions of AngII. The fact that human patients prescribed with a long-term ACE inhibitor or AngII receptor blocker therapy not only survive, but have improved clinical outcomes, also may minimize appreciation for the power of the day-to-day blood pressure actions of AngII. Our results show that, if not for the blood pressure actions of AngII, blood pressure would plummet ∼25 mmHg simply by switching to a LS diet for 7 days. That observation is not new (Davis et al. 1974; Hall et al. 1980), but what is new is the finding that this powerful effect of AngII is not dependent on IL-6. Chamarthi et al. (2011) reported that human subjects on LS diet did not have elevated plasma IL-6 levels. Notwithstanding, our results still were not necessarily predictable, because the degree to which blood pressure was under direct control of AngII (∼25 mmHg) is similar to what is reported for several models of AngII hypertension (Hall et al. 1980; Kawada et al. 2002; Gonzalez-Villalobos et al. 2009; Zhao et al. 2009; Brands et al. 2010). We recently reported an approximate 36 mmHg increase in MAP with chronic AngII infusion at 800 ng/kg per minute (Brands et al. 2010). Similarly, Zhang et al. (2012) reported approximately a 28 mmHg increase in MAP in WT mice infused with AngII at 1000 ng/kg per minute. Thus, with AngII having approximately a 20–30 mmHg blood pressure effect on LS diet, there was no reason on those grounds to predict that the physiologic role of AngII to prevent a decrease in blood pressure on LS diet should be independent of IL-6.

Our results showing no requirement for IL-6 on LS diet then forced the question of whether the range of blood pressure was important for determining whether IL-6 played a role in AngII-mediated blood pressure actions. Even though AngII was having a 20–30 mmHg blood pressure effect in the WT and IL-6 KO mice during LS diet, the failure to measure any dependence on IL-6 could have been because IL-6 only contributes to AngII-mediated blood pressure effects if blood pressure is increasing. Therefore, we tested whether IL-6 was required to mediate an AngII-mediated increase in blood pressure, but choosing an infusion rate of AngII that yielded plasma AngII levels not different than what was achieved physiologically in the mice on LS diet. That was necessary to separate the effect of plasma AngII concentration versus the blood pressure range itself. The challenge was to get a very low AngII infusion dose to cause 20–30 mmHg hypertension over a 7-day period.

Welch and Wilcox reported that AngII infusion at 200 ng/kg per minute s.c. in normal mice drinking saline caused approximately a 10 mmHg increase in MAP by day 9 (Kawada et al. 2002). We infused this dose in mice with ∼70% reduction in kidney mass (RKM) to enhance the ability of this very low AngII dose to cause hypertension. There was no effect of this AngII infusion dose in WT or KO mice with RKM until the switch from low- to high-salt diet (Fig.2). MAP increased ∼20 mmHg in both groups. Our analysis of plasma AngII concentration suggested that there was no difference in plasma AngII between the LS mice and the mice infused with AngII at this dose. Therefore, the combination of RKM and 4% high-salt diet enabled the plasma levels of AngII normally caused by LS diet in normal mice to cause 20–25 mmHg hypertension over 7 days. Moreover, this effect was not dependent on IL-6. Thus, whether AngII was providing ∼25 mmHg of physiologic blood pressure support on LS diet, or whether similar plasma levels of AngII were causing ∼25 mmHg increase in MAP, there was no requirement for IL-6. This suggests that the plasma level of AngII, not the elevated blood pressure per se, is a primary determinant of whether IL-6 is stimulated by AngII and is a factor in mediating the blood pressure actions of AngII.

Our signaling data not only are consistent with our previous reports (Brands et al. 2010; Banes-Berceli et al. 2011), but also show a difference between LS and AngII infusion at 200 ng/kg per minute. We reported that the activation of renal JAK2/STAT3 induced by 7 days of AngII hypertension at the infusion dose of 800 ng/kg per minute was prevented in IL-6 KO mice (Brands et al. 2010). Consistent with that, the present study also measured increased plasma IL-6 and JAK2/STAT3 phosphorylation in mice infused with that dose. Although we have not tested the role of JAK2/STAT3 on the hypertensive response in the mouse using the JAK2 inhibitor, AG-490, we did report in rats that there was a dose-dependent effect of AngII infusion to cause hypertension and JAK2/STAT3 phosphorylation, and AG-490 prevented the hypertension (Banes-Berceli et al. 2011). We also reported that rats on LS diet did not have increased JAK2/STAT3 phosphorylation, and that AG-490 had no effect on blood pressure (Banes-Berceli et al. 2011). Consistent with those findings, the present study measured no stimulation of JAK2/STAT3 phosphorylation in mice on LS diet. However, we did measure JAK2/STAT3 phosphorylation in mice infused with AngII at 200 ng/kg per minute, even though plasma AngII levels were not different from the mice on LS diet. The increase in serine and decrease in tyrosine phosphorylation of SHP-1 only in the two AngII-infused groups provides confidence that AngII was mediating a decrease in SHP-1 activity, which in turn supports the activation of JAK2/STAT3 only in those two groups. Thus, although the physiologic levels of AngII that occur with LS diet did not require IL-6 for blood pressure effects either above or below baseline, activation of JAK2/STAT3 was uniquely associated with AngII-mediated increases in blood pressure.

Although the blood pressure response of IL-6 KO mice to AngII infusion at 800 ng/kg per minute was not repeated in this study, these results together with our previous data (Brands et al. 2010) provide unique insights and add new information that help evaluate the role of inflammatory mechanisms over a wide range of AngII-mediated blood pressure effects. The AngII infusion dose of 800 ng/kg per minute (∼20 ng/min) we used recently (Brands et al. 2010) was approximately one-fourth of the infusion rate in our first study (90 ng/min; ∼3600 ng/kg per minute) (Lee et al. 2006), and the hypertension was completely blocked (Brands et al. 2010) – versus ∼50% attenuated (Lee et al. 2006) – in IL-6 KO mice. Similarly, Zhang et al. (2012) reported recently that AngII hypertension caused by an infusion dose of 1000 ng/kg per minute (∼25 ng/min) was almost completely prevented in IL-6 KO mice, whereas Ju et al. (2013) (2500 ng/kg per minute; ∼62 ng/min) and Gonzalez et al. (2015) (90 ng/min; ∼3600 ng/kg per minute) recently reported that AngII hypertension was not attenuated in IL-6 KO mice. Thus, very high AngII infusion doses appear to have non-IL-6-dependent AngII hypertensive actions, whereas at lower doses that may be more pathophysiologically relevant (800–1000 ng/kg per minute), virtually all the hypertensive action of AngII is dependent on IL-6 (Brands et al. 2010; Zhang et al. 2012). Activation of the intrarenal RAS has been proposed to facilitate the development of AngII-dependent hypertension. IL-6 contributes to augmentation of angiotensinogen expression in cultured renal proximal tubular cells (Satou et al. 2008, 2009). In AngII-infused mice, elevated intrarenal angiotensinogen was observed when mice received AngII infusion at 400 ng/kg per minute, although 1000 ng/kg per minute AngII infusion decreased intrarenal renin levels and failed to induce intrarenal angiotensinogen elevation (Gonzalez-Villalobos et al. 2008). On the other hand, Zhang et al. (2012) demonstrated that the intrarenal IL-6/ET-1 axis is associated with AngII-dependent hypertension. These results suggest that further diverse mechanisms are involved in IL-6-mediated blood pressure elevation in a dose-dependent manner even within the pathophysiological range of AngII infusion. Moving lower, to an AngII infusion dose (200 ng/kg per minute; ∼5 ng/min) designed to replicate plasma AngII levels that occur during LS intake, the present results show that the hypertensive action of AngII is not dependent on IL-6, nor is blood pressure maintenance on LS diet. JAK2/STAT3 signaling also is not required for blood pressure maintenance on LS diet, and appears uniquely linked to AngII hypertension, even at physiologic plasma AngII concentrations.

## References

[b1] Banes-Berceli AK, Hind AA, Proctor D, Qu H, Hill-Pryor C, Webb RC (2011). Angiotensin II utilizes Janus kinase 2 in hypertension, but not physiologic control of blood pressure. Am. J. Physiol. Regul. Integr. Comp. Physiol.

[b2] Brands MW, Banes-Berceli AK, Inscho EW, Al-Azawi H, Allen AJ, Labazi H (2010). Interleukin 6 knockout prevents angiotensin II hypertension: role of renal vasoconstriction and Janus kinase 2/signal transducer and activator of transcription 3 activation. Hypertension.

[b3] Chamarthi B, Williams GH, Ricchiuti V, Srikumar N, Hopkins PN, Luther JM (2011). Inflammation and hypertension: the interplay of interleukin-6, dietary sodium, and the renin-angiotensin system in humans. Am. J. Hypertens.

[b4] Crowley SD, Gurley SB, Herrera MJ, Ruiz P, Griffiths R, Kumar AP (2006). Angiotensin II causes hypertension and cardiac hypertrophy through its receptors in the kidney. Proc. Natl. Acad. Sci. USA.

[b5] Davis JO, Freeman RH, Johnson JA, Spielman WS (1974). Agents which block the action of the renin-angiotensin system. Circ. Res.

[b6] Fox J, Guan S, Hymel AA, Navar LG (1992). Dietary Na and ACE inhibition effects on renal tissue angiotensin I and II and ACE activity in rats. Am. J. Physiol.

[b7] Gonzalez GE, Rhaleb NE, D’Ambrosio MA, Nakagawa P, Liu Y, Leung P (2015). Deletion of interleukin-6 prevents cardiac inflammation, fibrosis and dysfunction without affecting blood pressure in angiotensin II-high salt-induced hypertension. J. Hypertens.

[b8] Gonzalez-Villalobos RA, Seth DM, Satou R, Horton H, Ohashi N, Miyata K (2008). Intrarenal angiotensin II and angiotensinogen augmentation in chronic angiotensin II-infused mice. Am. J. Physiol. Renal. Physiol.

[b9] Gonzalez-Villalobos RA, Satou R, Seth DM, Semprun-Prieto LC, Katsurada A, Kobori H (2009). Angiotensin-converting enzyme-derived angiotensin II formation during angiotensin II-induced hypertension. Hypertension.

[b10] Guzik TJ, Hoch NE, Brown KA, McCann LA, Rahman A, Dikalov S (2007). Role of the T cell in the genesis of angiotensin II induced hypertension and vascular dysfunction. J. Exp. Med.

[b11] Hall JE, Guyton AC, Smith MJ, Coleman TG (1980). Blood pressure and renal function during chronic changes in sodium intake: role of angiotensin. Am. J. Physiol.

[b12] Imig JD, Navar GL, Zou LX, O’Reilly KC, Allen PL, Kaysen JH (1999). Renal endosomes contain angiotensin peptides, converting enzyme, and AT(1A) receptors. Am. J. Physiol.

[b13] Ju X, Ijaz T, Sun H, Ray S, Lejeune W, Lee C (2013). Interleukin-6-signal transducer and activator of transcription-3 signaling mediates aortic dissections induced by angiotensin II via the T-helper lymphocyte 17-interleukin 17 axis in C57BL/6 mice. Arterioscler. Thromb. Vasc. Biol.

[b14] Kawada N, Imai E, Karber A, Welch WJ, Wilcox CS (2002). A mouse model of angiotensin II slow pressor response: role of oxidative stress. J. Am. Soc. Nephrol.

[b15] Kirabo A, Kearns PN, Jarajapu YP, Sasser JM, Oh SP, Grant MB (2011). Vascular smooth muscle Jak2 mediates angiotensin II-induced hypertension via increased levels of reactive oxygen species. Cardiovasc. Res.

[b16] Lee DL, Sturgis LC, Labazi H, Osborne JB, Fleming C, Pollock JS (2006). Angiotensin II hypertension is attenuated in interleukin-6 knockout mice. Am. J. Physiol. Heart Circ. Physiol.

[b17] Marrero MB, Venema VJ, Ju H, Eaton DC, Venema RC (1998). Regulation of angiotensin II-induced JAK2 tyrosine phosphorylation: roles of SHP-1 and SHP-2. Am. J. Physiol.

[b18] Mattson DL (2014). Infiltrating immune cells in the kidney in salt-sensitive hypertension and renal injury. Am. J. Physiol. Renal. Physiol.

[b19] McMaster WG, Kirabo A, Madhur MS, Harrison DG (2015). Inflammation, immunity, and hypertensive end-organ damage. Circ. Res.

[b20] Satou R, Gonzalez-Villalobos RA, Miyata K, Ohashi N, Katsurada A, Navar LG (2008). Costimulation with angiotensin II and interleukin 6 augments angiotensinogen expression in cultured human renal proximal tubular cells. Am. J. Physiol. Renal. Physiol.

[b21] Satou R, Gonzalez-Villalobos RA, Miyata K, Ohashi N, Urushihara M, Acres OW (2009). IL-6 augments angiotensinogen in primary cultured renal proximal tubular cells. Mol. Cell. Endocrinol.

[b22] Shao W, Seth DM, Prieto MC, Kobori H, Navar LG (2013). Activation of the renin-angiotensin system by a low-salt diet does not augment intratubular angiotensinogen and angiotensin II in rats. Am. J. Physiol. Renal. Physiol.

[b23] Singh MV, Chapleau MW, Harwani SC, Abboud FM (2014). The immune system and hypertension. Immunol. Res.

[b24] Zhang J, Crowley SD (2015). Role of T lymphocytes in hypertension. Curr. Opin. Pharmacol.

[b25] Zhang W, Wang W, Yu H, Zhang Y, Dai Y, Ning C (2012). Interleukin 6 underlies angiotensin II-induced hyper-tension and chronic renal damage. Hypertension.

[b26] Zhao D, Seth DM, Navar LG (2009). Enhanced distal nephron sodium reabsorption in chronic angiotensin II-infused mice. Hypertension.

